# Racial inequalities in access to healthcare services in Brazil (2019): a decomposition analysis

**DOI:** 10.1186/s12913-025-13527-6

**Published:** 2025-12-05

**Authors:** Rony Coelho, Matías Mrejen, Lucas Falcão, Rudi Rocha, Thomas Hone

**Affiliations:** 1 Instituto de Estudos para Políticas de Saúde, São Paulo, Brazil; 2 Municipal Government of São Paulo, São Paulo, Brazil; 3 René Rachou Institute, Fundação Oswaldo Cruz (Fiocruz), Belo Horizonte, Brazil; 4 São Paulo School of Business Administration (FGV EAESP), São Paulo, Brazil; 5 Public Health Policy Evaluation Unit, School of Public Health, Imperial College London, London, UK

**Keywords:** Racial inequalities, Healthcare access, Socioeconomic factors, Discrimination

## Abstract

**Background:**

Racial and ethnic inequalities in access to healthcare services pose a significant challenge for many countries, with ethnic minorities and Afro-descendants frequently experiencing poorer access to services and health outcomes. However, little is known about the factors that drive racial inequalities in access to healthcare, especially in low- and middle-income countries.

**Methods:**

This is a cross-sectional, observational study that uses data from the National Health Survey (2019) in Brazil and assesses the contribution of predisposing, facilitating, and need-for-care factors to inequalities in healthcare access between White, Black and Pardo (Brown) Brazilians. The Oaxaca-Blinder method was used to separate differences between racial groups into explained and unexplained determinants. Variables included in the models were age, sex, marital status, family size and composition, access to sanitation, health insurance, primary care coverage, income quintile, employment status, education, and self-rated health status.

**Results:**

The responses from 204,918 individuals were analysed. White Brazilians reported better access to healthcare and medications than Black or Pardo Brazilians, with the largest racial inequalities in unmet needs for healthcare services and access to medication. Racial inequalities are mostly explained by observable factors (between 35% and 87%), most notably the access to financial resources (income and access to private health insurance). Being covered by the Family Health Program and higher levels of education were associated with reduced inequalities between racial groups. The unexplained portion of inequalities was the greatest when comparing Black and White individuals, implying that factors beyond socioeconomic status likely drive these inequalities including community, cultural or behavioural factors, and inequalities and bias in healthcare services.

**Conclusions:**

While investments in universal healthcare and education may be important for reducing racial inequalities in access to healthcare, better understanding of cultural and contextual factors and targeted public policies and services interventions for addressing racial inequalities are needed.

**Supplementary Information:**

The online version contains supplementary material available at 10.1186/s12913-025-13527-6.

## Background

Racial and ethnic inequalities in access to healthcare services are a major challenge for both high-income [1–7] and low- and middle-income countries (LMICs) [8–11] These inequalities are often large and unjust [12]. Inequalities in access to healthcare often stem from certain ethnic and racial groups experiencing lower socioeconomic status and discrimination [13]. These factors include barriers such as lower incomes and educational opportunities, unfavourable geographic location, precarious work, and greater exposure to environmental risks. However, racial and ethnic inequalities in healthcare access are not always fully explainable by these socioeconomic factors. Race and ethnicity are important social determinants of health themselves, as they are related to trust in providers, healthcare-seeking behaviours, discrimination and prejudice or racism [14–16].

Unequal access to healthcare directly contributes to inequality in health outcomes. Healthcare access refers to an individual’s capability to acquire healthcare services for the prevention, diagnosis, treatment, and management of diseases and health conditions, and depends on service availability, affordability, accessibility, and acceptability. These different aspects of access vary across for socioeconomic groups, including racial and ethnic groups, and assessments of access must encompass an understanding of different health needs, as well as socioeconomic and cultural factors [17].

A wealth of studies document large inequalities in access to healthcare across ethnic and racial groups. However, much of this evidence comes from high-income countries. In the United States (US), compared to non-Hispanic White individuals, African Americans and Hispanics have lower insurance coverage rates [18]. Large inequalities exist in access to medication in the US, for example, for treating opioid use disorders [19]. In England and Wales, while stillbirth and infant mortality rates have decreased since 2007 for various ethnic groups, infant mortality is still higher among ethnic minorities [12]. In LMICs, there are large racial inequalities in access to health insurance for children – for example in Argentina, Brazil, and Ecuador [8]. In South Africa, Black individuals who sought outpatient care spent substantially higher transport costs than White individuals [20].

Brazil is a relevant case for examining racial inequalities in access to healthcare services. There is a wealth of literature documenting racial inequalities in health outcomes in the country [21], where Black and Pardo (a term used in Brazil to refer to people of mixed race, and sometimes also translated as Brown) Brazilians are disadvantaged. For example, Black and Pardo Brazilians have higher rates of anxiety and depression [22]. Children of Black mothers have a 72% higher risk of dying from diarrhoea compared to children of White mothers [23]. Breast cancer mortality rates are higher among Black than among White women [24]. Black or Pardo individuals are more likely to have untreated depression [25]. However, despite evidence of racial inequalities in health outcomes and healthcare access in Brazil, little is known about what factors contribute to these inequalities.

Using data from the National Health Survey (2019), we analyse racial inequalities in access to health services in Brazil, quantifying the role of predisposing factors (e.g., age, gender), enabling factors (e.g., income, access to private health insurance), and need for health factors (e.g., health status). This classification is based on Andersen’s model [26], which focuses on the behavioural model of health services utilization. We consider four measures of access and compare differences between White and Black Brazilians and between White and Pardo Brazilians: (i) unmet need for healthcare; (ii) unmet need for medication; (iii) inability to receive healthcare last time they sought; and (iv) people diagnosed with a chronic disease who have not received medical care in the last year. We use the Oaxaca-Blinder method, which is widely used in the literature for this purpose [25, 27, 28]. This method decomposes the differences between two groups into explained (such as education and income) and unexplained components (such as discrimination). This allows us not only to quantify inequalities but also identify which factors contribute to these inequalities and the magnitude of those contributions. The unexplained component of the decomposition method is important in studies assessing racial and ethnic inequalities as a large and persistent unexplained component might indicate specific race-related factors such as cultural differences, higher levels of chronic stress experienced by racial minorities [29], bias by health professionals, trust in providers, structural differences in access and quality of healthcare, and discrimination [30]. These conclusions have been drawn by a wide range of existing studies in other topics or settings [25, 31–34].

## Methods

This is a cross-sectional study that employs the Oaxaca-Blinder decomposition to estimate the explained and unexplained parts of racial differences in access to healthcare between White individuals and non-White individuals in Brazil. For the explained component, we also analyse explanatory factors that exacerbate or mitigate these disparities.

### Data

We analysed data from the 2019 Brazilian National Health Survey (Pesquisa Nacional de Saúde, PNS). The survey was conducted jointly by the Brazilian Institute of Geography and Statistics (*Instituto Brasileiro de Geografia e Estatística* (IBGE)) and the Ministry of Health [35]. The survey includes a wide range of socioeconomic characteristics and data on health status, as well as on the use of healthcare services by all individuals in sampled households. The data are publicly accessible and anonymized. Ethical principles of transparency, privacy, confidentiality, and protection of the individual rights of participants are therefore respected.

### Variable of interest (exposure)

Self-reported race/ethnicity is the main variable of interest. In Brazil, the official racial classification is determined by an individual’s self-declaration and is based on skin colour, specifically Black, Pardo, White, Indigenous, and Yellow (Asian). However, beyond skin color, identification is usually based on observable attributes such as hair texture, facial structure, and cultural and ancestry [36]. For this study, we employed two sets of racial pairings: Black vs. White and Pardo vs. White. In the appendix we also report the comparison between Black and Pardo individuals. Although the aggregation of Black and Pardo groups is common in Brazil, we analyse these groups separately, as studies show that Black individuals experience worse health outcomes compared to Pardo individuals [24, 37]. In 2022, 45% of the Brazilian population self-identified as Pardo, 10.2% as Black, and 43.5% as White. We omitted Indigenous (0.6%) and Yellow populations (0.4%) from this study due to low numbers and the specificities of these populations [36].

### Dependent variables (outcomes)

We used four dependent variables to evaluate healthcare access (see Appendix), which align with widely accepted definitions of access [17]: (i) unmet need for healthcare; (ii) unmet need for medication; (iii) inability to obtain services the last time healthcare was sought; and (iv) unmet need for healthcare for individuals diagnosed with a chronic disease (unmet need for chronic care).

Unmet need for healthcare was based on the survey question asking respondents who sought health care in the past two weeks. Those who sought healthcare but were denied on the first attempt or those who did not seek care when needed were defined as having unmet healthcare needs. Unmet need for medication was defined as respondents who were unable to get all their medications prescribed in their last doctor visit. The third dependent variable is inability to obtain services the last time healthcare was sought. It was defined as individuals who did not receive healthcare during their last healthcare visit. Those who received care were categorized as not experiencing this unmet need. Finally, the fourth variable identifies individuals diagnosed with chronic diseases who have not received medical care within the past year. Responses were obtained from survey questions querying the duration since the last medical consultation of the individuals interviewed, and if they were ever diagnosed by a physician with a chronic disease. Those diagnosed with a chronic disease who reported not having visited a doctor within the past year, regardless of the reason, were categorized as experiencing an unmet need for medical care.

### Independent variables

We categorized the independent variables into three dimensions: predisposing factors, enabling factors, and need-for-care factors using Andersen’s behavioural model of health services use [26]. We used a literature review [38] of common variables for the three dimensions to define the independent variables and categorize them into dimensions of access.

For predisposing factors, we assessed age (18–24 years, 25–34, 35–44, 45–54, 55–64, 65 or more), sex (men/women), marital status (married, divorced, single, widower), family size (number of family member), the presence of an elderly family member in the household (yes/no), and household’s access to basic sanitation (yes/no).

For enabling resources factors, we separated them into: (a) financial resources, health insurance and primary care coverage, where we assessed registration with family health teams (FHT) (yes/no), coverage of private health insurance (yes/no), and income quintiles; and (b) other enabling factors, where employment status (employed, unemployed, and out of the workforce) and education level (none, low, medium, or highest level), internet availability (yes/no), urban residency (yes/no), and slum proxy (yes/no) were included. We included access to FHTs due to importance of Brazil’s Family Health Strategy (FHS). The FHS is Brazil’s flagship primary care program, delivering comprehensive, community-based healthcare through multidisciplinary teams [39]. Expansion of the FHS has been strategically targeted toward lower-income and underserved areas, where Black and Pardo populations are disproportionately represented, and evidence suggests the FHS has contributed to reducing racial inequalities in healthcare access by improving service availability and continuity for historically marginalized groups [9, 40]. We included private health insurance as it facilitates access to private healthcare services and is associated with higher utilisation in Brazil [41].

The third dimension, need for care factor, encompassed variables related to health status, including interruption of usual activities due to health reasons (yes/no) and perceived health status (very bad, bad, regular, good, and very good).

### Statistical analysis

First, we calculated the percentages of each dependent variable for each racial group in order to compare the differences in rates across racial pairings (Black vs. White and Pardo vs. White (and Black vs. Pardo in the Appendix)). We calculated the mean percentages of the independent variables for each group. Survey weights were used in all analyses to adjust for the sampling design of the PNS and ensure representativeness of the results to the entire Brazilian population.

To assess the contribution of different factors to racial disparities in healthcare access, we employed the Oaxaca-Blinder decomposition method. This technique decomposes the mean difference in outcomes between two groups into explained and unexplainable components. The explained component is attributable to differences in observable characteristics, whilst the unexplained component can reflect other non-measured variables such other socioeconomic factors, discrimination, cultural differences, or structural inequalities. The method is based on a counterfactual framework, estimating what the outcome for one group would be if they had the same characteristics as the other group [28, 30].

In all decomposition models, we included a comprehensive set of covariates informed by Andersen’s behavioural model of health services use (see above), grouped into predisposing, enabling, and need-for-care factors. The covariates allowed us to adjust for confounding whilst also serving as potential explanatory variables within the explained component of the decomposition. The Oaxaca-Blinder method assumes linearity in the relationship between covariates and outcomes, correct model specification, and that all relevant variables are included.

All statistical analyses were conducted using Stata 17 (StataCorp LLC, College Station, TX), which includes built-in commands for Oaxaca-Blinder decomposition.

## Results

The study sample contained 204,918 respondents aged 18 or older, of which 75,376 (36.8%) identified as White, 22,864 (11.2%) as Black, and 106,678 (51.2%) as Pardo (Table 1). White respondents were, on average, slightly older than Black and Pardo individuals, more likely to be married or divorced, and had higher access to adequate sanitation. No significant disparities were observed in gender distribution or household size across racial groups, although households with elderly members were more common among White respondents. Income quintile and other enabling factors related to socioeconomic status revealed substantial inequalities. White respondents had the highest rates of higher education attainment—29% compared to roughly 14% among Black and Pardo individuals—and were disproportionately represented in the highest income quintiles, while Black and Pardo respondents were more likely to reside in areas classified as slums. Enabling factors related to health insurance and primary care coverage also varied considerably. While Black and Pardo respondents had higher rates of registration with family health teams (FHTs), White individuals had significantly higher rates of private health insurance coverage. Factors related to the need for care followed a similar gradient. White respondents were more likely to rate their health as “very good” and less likely to report interruption in daily activities due to health issues.

**Fig. 1:**
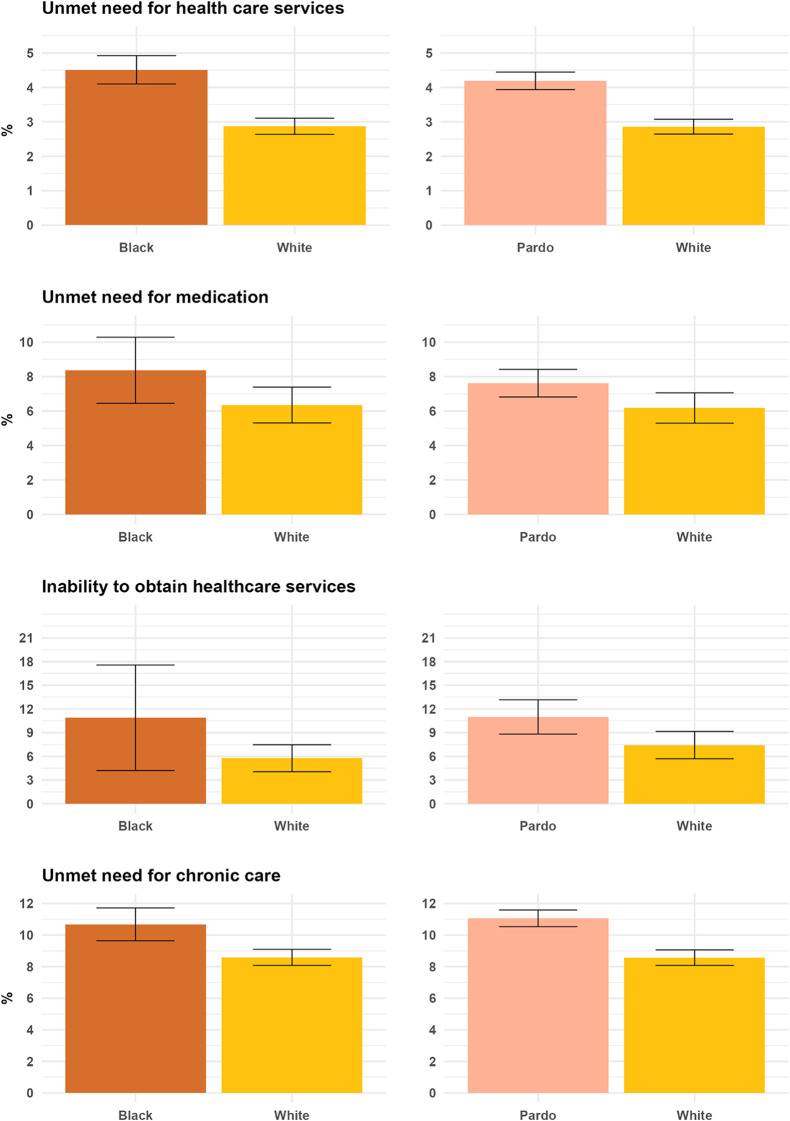
Levels of access across four dimensions by racial groups. Note: The figure displays the differences between White and Black individuals (left column), and between White and Pardo individuals (right column), across four indicators of access to healthcare. Error bars represent 95% confidence intervals. All estimates are weighted to account for the complex sampling design. Levels of access among White individuals can differ between the two comparisons due to survey design considerations. For valid variance estimation, the analysis effectively focuses on strata and primary sampling units containing both White individuals and the respective comparison group (Black or Brown individuals). This can result in different effective samples of White individuals being used in each comparison

Racial disparities in access to healthcare were assessed across four outcome indicators (Fig. 1), with Black and Pardo respondents consistently reporting worse access across all four measures compared to White respondents. Black respondents also showed worse levels of access than Pardo respondents in all measures, except for unmet need for chronic care (see Appendix); however, these differences were generally not statistically significant.

For unmet need for healthcare services, 4.5% (95% CI: 4.1–4.9) of Black individuals and 4.2% (95% CI: 3.9–4.4) of Pardo individuals reported having unmet needs for healthcare, compared to only 2.95% (95% CI: 2.6–3.1) among White individuals. For unmet need for chronic care, 10.7% (95% CI: 9.6–11.7) of Black respondents and 11.1% (95% CI: 10.5–11.6) of Pardo respondents reported unmet needs, compared to 8.6% (95% CI: 8.1–9.1) of White individuals. This was the only dimension where Pardo respondents had slightly worse outcomes than Black respondents, though the difference was not statistically significant. There were also gaps for unmet need for medications and inability to obtain healthcare services between Black/Pardo and White respondents, although these were nonsignificant.

**Fig. 2:**
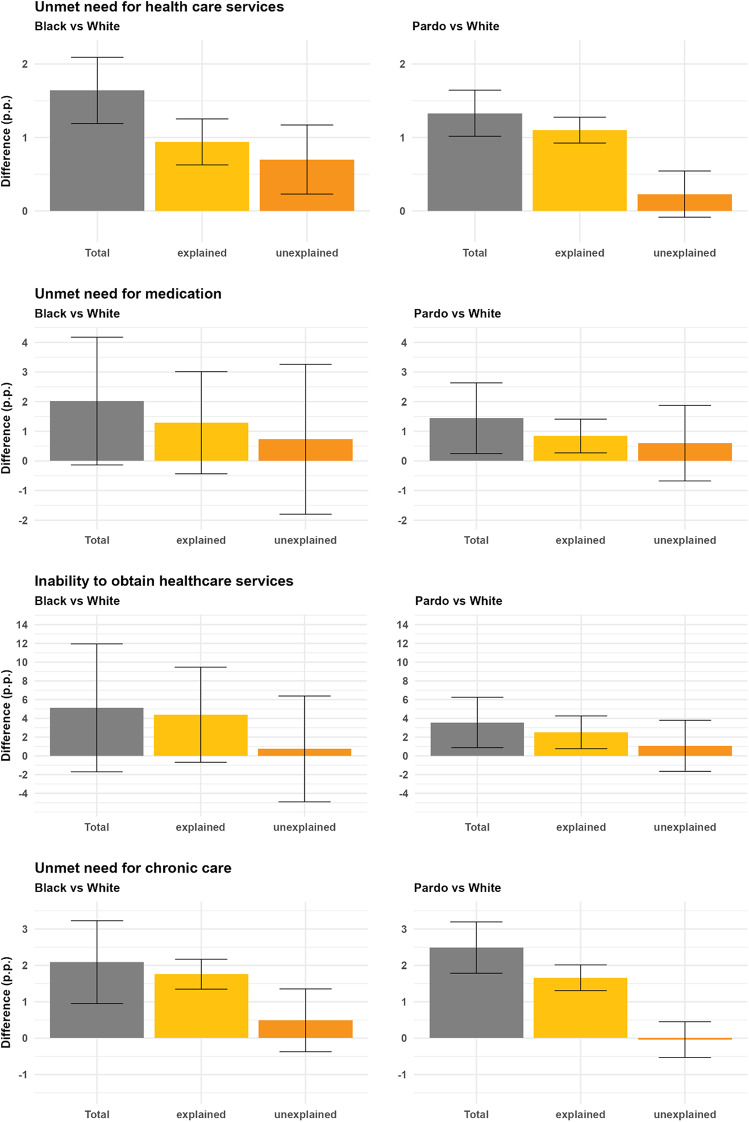
Results from Oaxaca-Blinder decompositions. Note: The figure presents results from an Oaxaca-Blinder decomposition of differences between White and Black individuals (left column), and between White and Pardo individuals (right column), across four indicators of access to healthcare. In each case, the difference is decomposed into an explained component, attributable to mean differences in observable characteristics between groups, and an unexplained component. Error bars represent 95% confidence intervals

In Oaxaca-Blinder decompositions, across all four outcomes and for both racial pairings (Black vs. White and Pardo vs. White), a substantial share of the observed disparities was explained by observable variables (Fig. 2). For unmet need for healthcare, 56.2% of the observed difference between Black and White individuals was explained by observable variables (explained difference: 0.9% point (p.p.) (95% CI: 0.6–1.3); total difference: 1.6 p.p. (95% CI: 1.2–2.1)). For Pardo and White individuals for the same outcome, explained factors accounted for 84.6% of the total observed difference (total difference: 1.3 p.p. (95% CI: 1–1.6); explained difference 1.1 p.p. (95% CI: 0.9–1.3)).

For the difference in unmet need for medication between the Black and White respondents, 65% of the difference was explained by observed variables (total difference: 2 p.p. (95% CI: -0.1–4.2); explained difference 1.3 p.p. (95% CI: -0.4–3)). Similarly, the differences between Black and White respondents in the inability to obtain healthcare services when last sought and in unmet needs for chronic disease were 13.7% and 21.7% respectively.

**Fig. 3:**
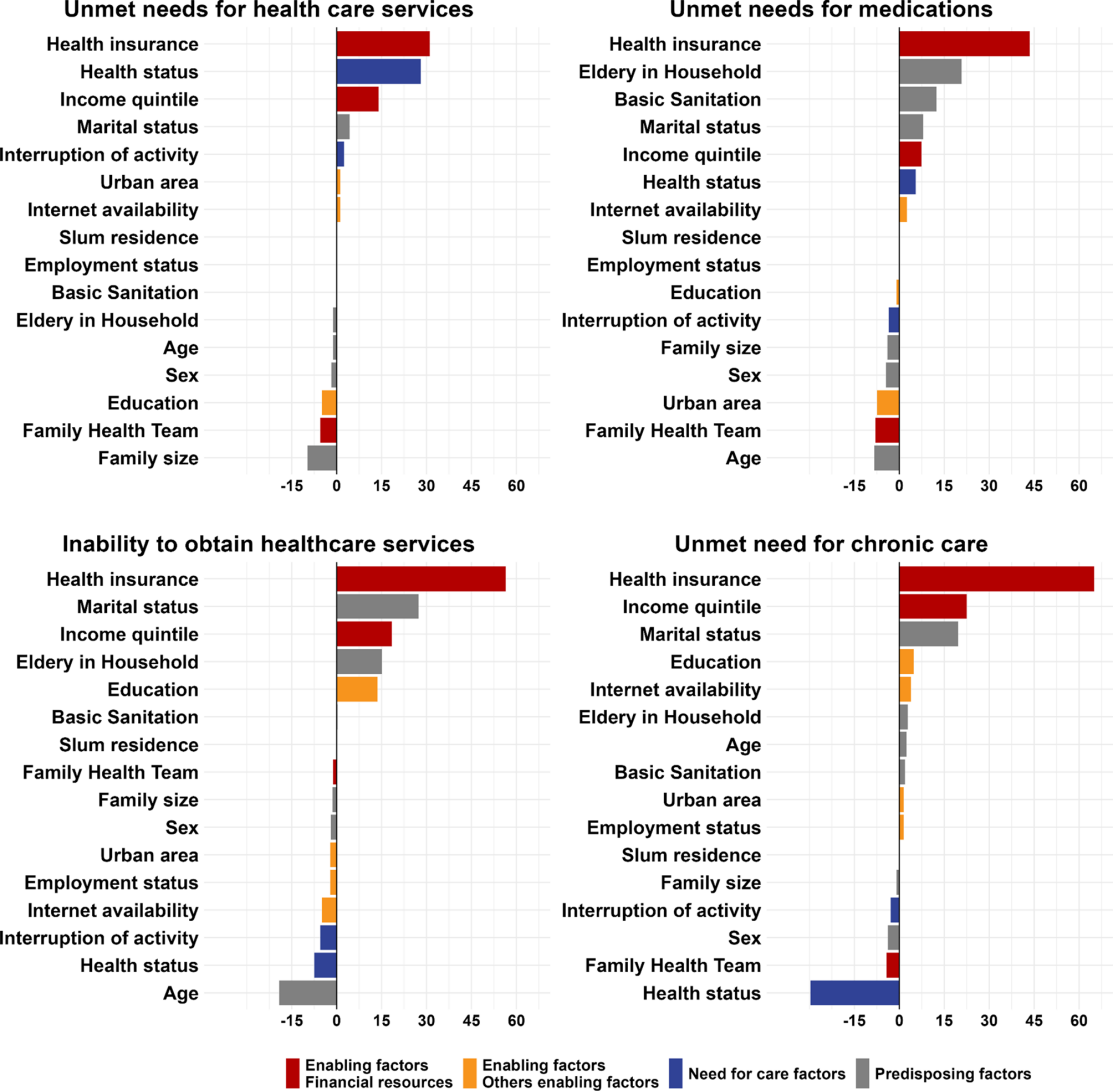
Contributions to total difference of the explained part: Black vs. White. Note: The figure presents results from an Oaxaca-Blinder decomposition of differences between White and Black individuals. The bars represent the percentual contribution of each contributing factor to the explained part in the corresponding decomposition presented in Fig. 2. For covariates with more than one category, the bars display the sum of the percentual contribution of all categories. All reported data are weighted considering the sampling design

**Fig. 4:**
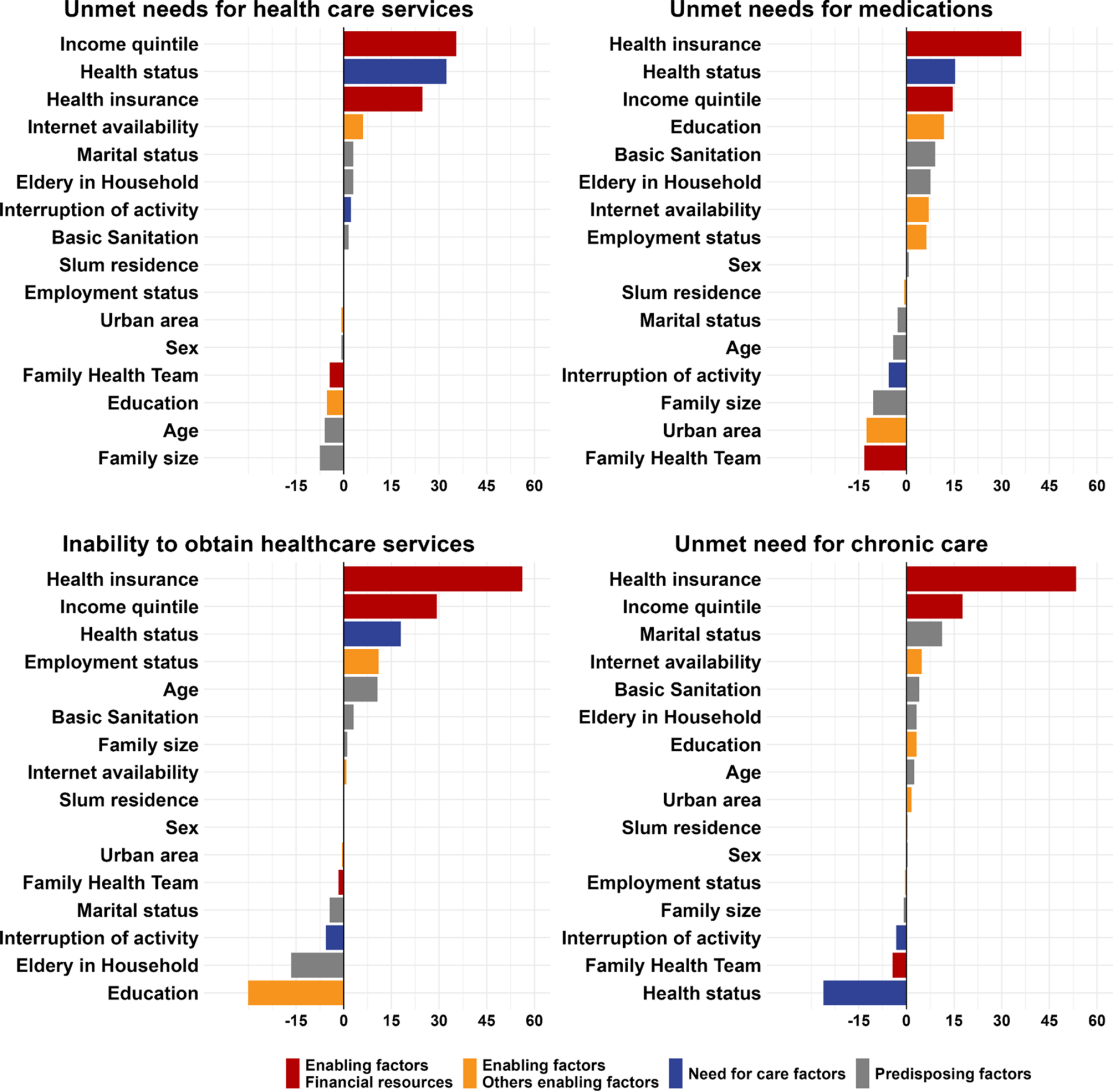
Contribution to total difference of the explained part: Pardo vs. White. Note: The figure presents results from an Oaxaca-Blinder decomposition of differences between White and Pardo individuals. The bars represent the percentual contribution of each contributing factor to the explained part in the corresponding decomposition presented in Fig. 2. For covariates with more than one category, the bars display the sum of the percentual contribution of all categories. All reported data are weighted considering the sampling design

Figures 3 and 4 display the contribution of individual factors to the explained portion of racial disparities in healthcare access between Black and White individuals (Fig. 3), and between Pardo and White individuals (Fig. 4). Across both comparisons and all four access indicators, the variables which were the most important contributors to the explained component were financial resources, access to private health insurance, income quintile, primary care coverage, and private health insurance.

In the comparison between Black and White individuals (Fig. 3), private health insurance was the dominant contributor to racial disparities in access to healthcare across all four outcomes. It accounted for over half of the explained portion in the cases of inability to obtain healthcare services when last sought (55.1%) and unmet need for chronic care (65.1%). For unmet need for medications, it explained 43.1% of the disparity, and for unmet need for healthcare services, over 30%. Income quintile was also a major contributor, explaining over 10% of the disparity in unmet need for healthcare, around 22% in unmet need for chronic disease care, and over 15% in inability to obtain healthcare services when last sought. Health status also played a significant role, accounting for nearly 30% of the explained portion of disparities in unmet need for healthcare services. Conversely, certain factors contributed consistently to reducing racial inequalities in access. Across all four outcomes, age and registration with a Family Health Team (FHT) were the most notable. For instance, in the case of unmet need for medication, FHT registration contributed to an 8.4% reduction in the racial access gap.

Patterns observed in the comparison between Pardo and White individuals (Fig. 4) largely mirrored those found in the Black vs. White comparison. Private health insurance emerged as the most significant driver of explained disparities in three of the four outcomes - accounting for over half of the explained portion in the decomposition for inability to obtain healthcare services when last sought (56.2%) and unmet need for chronic care (53.4%), as well as over one third (36.1%) of the explained component in the decomposition for unmet need for medication. Income quintile was also a significant contributor across all outcomes, accounting for 9.1% of the explained inequality in unmet healthcare needs, 14.6% in unmet need for medication, 29.2% in inability to obtain services when last sought, and 17.7% in unacquired care for chronic diseases. Health status again played an important role, contributing 32.8% to the explained disparities in unmet healthcare needs (32.8%), and around 15% in unmet needs for medications and inability to obtain care when last sought. As in the previous comparison, registration with a FHT also emerged as an important factor contributing to the reduction of racial inequalities in access.

**Table 1:** Summary statistics for independent variables

Independent variable	Black	Pardo	White
**Predisposing factors**			
Age (%)			
18–24	13.9 (13.2, 14.7)	15.5 (15.2, 15.9)	12.2 (11.9, 12.7)
25–34	19.6 (18.7, 20.5)	20.3 (19.9, 20.8)	17.3 (16.8, 17.8)
35–44	20.8 (20.0, 21.7)	20.7 (20.3, 21.2)	18.6 (18.2, 19.2)
45–54	18.0 (17.3, 18.9)	17.6 (17.2, 18.1)	18.0 (17.6, 18.5)
55 − 54	13.9 (13.2, 14.7)	13.2 (12.9, 13.7)	16.1 (15.7, 16.7)
65 or older	13.6 (12.9, 14.4)	12.4 (12.0, 12.8)	17.5 (17.0, 18.1)
Men (%)	48.4 (47.5, 49.4)	47.4 (47.1, 47.8)	45.8 (45.4, 46.3)
Marital Status (%)			
Married	36.9 (35.8, 38.1)	39.6 (39.0, 40.3)	46.6 (45.9, 47.4)
Divorced	6.2 (5.65, 6.87)	6.2 (5.98, 6.54)	7.8 (7.47, 8.16)
Single	50.4 (49.3, 51.7)	48.3 (47.7, 48.9)	38.3 (37.7, 39.0)
Widower	6.3 (5.8, 6.8)	5.81 (5.5, 6.0)	7.2 (6.8, 7.5)
Family size	3.3 (3.3, 3.4)	3.4 (3.5, 3.5)	3.1 (3.1, 3.1)
Presence of the elderly in the household (%)	19.6 (18.8, 20.5)	18.3 (17.9, 18.9)	25.0 (24.4, 25.7)
Household access to basic Sanitation (%)	85.8 (84.7, 86.9)	81.2 (80.5, 82.1)	88.6 (88.0, 89.3)
**Enabling factors**			
**a) Financial resources**			
Registered with FHT (%)	63.7 (62.1, 65.4)	67.0 (66.0, 68.0)	55.6 (54.1, 57.3)
Health insurance (%)	18.9 (17.9, 20.0)	18.4 (17.9, 19.1)	37.1 (36.0, 38.3)
Income quintile (%)			
1	19.7 (18.7, 20.9)	21.9 (21.3, 22.5)	8.6 (8.20, 9.12)
2	21.7 (20.6, 22.9)	22.3 (21.8, 23.0)	12.9 (12.4, 13.6)
3	23.6 (22.4, 24.8)	22.6 (22.0, 23.2)	18.8 (18.2, 19.6)
4	21.1 (20.0, 22.3)	19.4 (18.9, 20.1)	25.0 (24.3, 25.9)
5	13.6 (12.7, 14.7)	13.5 (13.0, 14.1)	34.3 (33.2, 35.5)
**b) Others enabling factors**			
Employment status (%)			
Employed	62.0 (60.9, 63.1)	58.8 (58.3, 59.4)	61.7 (61.0, 62.4)
Unemployed	6.5 (6.0, 7.1)	6.5 (6.3, 6.8)	4.2 (4.0, 4.5)
Out of the workforce	31.4 (30.4, 32.4)	34.6 (34.0, 35.1)	33.9 (33.3, 34.6)
Education (%)			
No Instruction	8.3 (7.7, 9.0)	8.1 (7.8, 8.4)	4.2 (3.9, 4.4)
Low	38.9 (37.8, 40.1)	39.6 (39.0, 40.2)	31.9 (31.1, 32.7)
Medium	38.4 (37.3, 39.5)	37.8 (37.2, 38.4)	34.4 (33.7, 35.2)
High	14.2 (13.4, 15.2)	14.4 (13.9, 14.9)	29.4 (28.4, 30.5)
Household internet availability (%)	82.2 (81.2, 83.2)	80.6 (80.1, 81.3)	87.5 (87.1, 88.1)
Urban (%)	86.5 (85.5, 87.5)	82.5 (82.0, 83.1)	89.5 (89.0, 90.0)
Slum proxy (%)	23.6 (22.2, 25.1)	27.8 (26.9, 28.8)	16.4 (15.6, 17.3)
**Need for care factors**			
Interruption of usual activities due to health reasons (%)	8.8 (8.2, 9.4)	8.6 (8.3, 8.9)	8.1 (7.8, 8.4)
Perceived health status (%)			
Very bad	1.3 (1.1, 1.6)	1.2 (1.1, 1.3)	1.0 (0.9, 1.1)
Bad	5.8 (5.3, 6.3)	5.2 (5.0, 5.5)	3.7 (3.5, 4.0)
Regular	28.5 (27.5, 29.6)	29.6 (29.1, 30.2)	22.6 (22.0, 23.2)
Good	51.5 (50.3, 52.8)	51.6 (51.0, 52.3)	54.0 (53.3, 54.7)
Very Good	12.6 (11.8, 13.6)	12.1 (11.7, 12.7)	18.5 (17.8, 19.3)

Note: The table presents the percentages and 95% confidence intervals for all variables used in the decomposition analysis, disaggregated by racial groups. All estimates are weighted to account for the complex sampling design

## Discussion

This study finds large racial inequalities in access to healthcare, mainly between Black or Pardo and White Brazilians. White individuals consistently show better access to healthcare compared to Black and Pardo Brazilians. For example, only 2.95% of White respondents reported an unmet need for healthcare compared to 4.5% of Black respondents and 4.2% of Pardo respondents. Most of this inequality is explained by observable characteristics, with income and access to private health insurance the largest contributors to inequalities. On the other hand, registration with primary care contributed to reductions in racial inequalities in access to healthcare. A sizeable component of racial inequalities in healthcare access persisted suggesting the importance of unobserved factors, mainly when comparing White and Black Brazilians. The literature frequently attributes these unexplained factors in differences between racial groups to systemic discrimination or institutional racism [33, 34, 42].

The presence of racial inequalities, with White Brazilians having greater levels of access than Black or Pardo Brazilians, is unsurprising and aligns with various recent studies [10, 37, 43–45]. A systematic analysis found that factors undermining the Black population’s access to health services include institutional and structural factors, such as poor infrastructure, service quality, and socioeconomic conditions [43]. Despite the increase in primary care coverage in Brazil, Black individuals have greater difficulty in accessing services [45].

We find Black and Pardo Brazilians are similarly disadvantaged in terms of access, yet the wider evidence base is ambiguous. In many studies, these racial categories are combined [45, 46], due to historical similarities in socioeconomic conditions and access to services [21]. However, in some cases when analysed separately, distinctions are found between Blacks and Pardos. For example, Black women had a much higher maternal mortality rate than White or Parda women in all age groups and for all causes [37]. Breast cancer mortality rates were higher among Black compared to White women [24]. Differences between Black and Pardo Brazilians may be expected as evidence demonstrates individuals with darker skin experience higher rates of discrimination [47, 48].

This study found that income and private health insurance were major drivers of racial inequalities. Together, they accounted for between 35% and 87% of the explainable part of the racial inequalities in healthcare access. Notably, White Brazilians are more likely to be in the highest income quintile, whereas only about 18% of Black and Pardo individuals have private health insurance, compared to 37% among White individuals. These findings highlight the structural role of income and private insurance in shaping access to healthcare. Importantly, registration with the FHT program was found to contribute to reductions in racial inequalities in access in this study. This may be because expansion of the FHS has been targeted towards lower income populations (who are more likely to be Black or Pardo) and aims to provide accessible community-based primary care. This aligns with other research finding FHS expansion disproportionately benefitted Black and Pardo Brazilians [9].

Despite identifying multiple explanatory factors for these racial inequalities, there remained a sizable unexplainable component. This is driven by factors that we were not able to measure in this study. There are likely many socioeconomic factors that may explain these findings, including wealth, occupation, social capital, neighbourhood environment, and educational quality. Distance to healthcare services is an important factor, with evidence showing Black/Pardo Brazilians travel greater distances for medium- and high-complexity services [46]. A common interpretation in the literature of the unexplainable component in racial inequalities is related to discrimination against those with darker skin and African ancestry - particularly racial bias in healthcare and institutional racism [49, 50]. Racial disparities in healthcare access are often attributed to a combination of structural and institutional factors [14, 15]. Black and Pardo Brazilians often reside in segregated areas, with fewer healthcare facilities and where healthcare quality may be lower [51]. Studies show even after controlling for health insurance and socioeconomic status, racial minorities in the US still receive lower-quality healthcare compared to White individuals [13]. In Brazil, darker-skinned women receive lower quality care during pregnancy and childbirth [50]. Experiences of discrimination and racism are associated with negative patient experiences, including lower levels of healthcare-related trust, satisfaction, and communication, and poorer health service use such as delayed used and lower adherence to treatment [52].

One limitation of this study is the use of cross-sectional data, which prevents establishing causal relationships over time. While the Oaxaca-Blinder decomposition effectively quantifies disparities, it has its limitations - it is limited in its ability to capture complex, non-linear relationships and interactions between variables. The decomposition method also assumes the model is correctly specified and all relevant covariates are included. The unexplained component may be conflated by measurement error, omitted variables, and structural inequalities meaning caution is need in interpretation. A more in-depth analysis could explore the possibility of complementing this study with qualitative approaches to capture cultural and contextual nuances that quantitative data may not fully address. Additionally, some relevant variables, such as other socioeconomic factors, cultural factors, discrimination and racism, could not be directly included in the decomposition. Racial classification in Brazil is also complex and is based on self-identification, which may result in variations in how individuals perceive and report their racial identity. There is likely to be sizeable bias in how different populations are reporting their skin colour and this may correlate with socioeconomic factors, discrimination and treatment by other people. There is a historical trend in Brazil for individuals to report a lighter skin colour than what may be observed, and this misclassification bias may be reducing the ability of this study to detect and explain inequalities. However, it is worth noting that evidence suggests that historical trend has shifted in recent years [53]. The results of this study should be complemented with further qualitative work to better understand racial inequalities in healthcare access, and future research should better assess how bias from self-classification across racial groups could affect inequalities in healthcare access.

Our findings imply that racial inequalities in access to healthcare require multifaceted and intersectoral solutions [12], addressing both socioeconomic factors, race group-specific factors, and healthcare provision. Private health insurance was a major contributor to racial inequalities in healthcare access, however further expanding private health insurance may exacerbate inequities given the costs and negative impacts on the public system (SUS). Public investments in universal access to healthcare, including the FHS are important for addressing racial inequalities [54]. Efforts to increase access of healthcare, regardless of income or educational attainment are important. For example, there are targeted interventions for stroke prevention [55] and cancer treatment for ethnic minorities [56]. The healthcare workforce should better reflect the ethnic and cultural diversity of the communities they serve, ensuring culturally appropriate communication and informational materials. Training in cultural competence and patient-centered communication is important. Monitoring the quality of care, stratifying data by race and ethnicity, and collecting patient feedback are also essential.

Brazil’s National Policy for the Integral Health of the Black Population aims to promote health equity for the Black population by addressing historical and structural inequalities. It seeks to ensure adequate access to healthcare services, promote prevention and treatment actions specific to health conditions prevalent among the Black population, and encourage the training of healthcare professionals to address issues of race and ethnicity. However, there is little documentation of the implementation of these measures or analysis of this policy over time, and it remains to be seen if the policy has reduced disparities and improved the health of the Black population [57].

## Conclusion

Significant racial disparities in healthcare access in Brazil exist, with Black and Pardo individuals facing greater barriers compared to their White counterparts. These disparities are largely driven by observable factors, such as income and private health insurance. The findings underscore the need for targeted public policies to address these disparities and promote equity in healthcare access.

## Supplementary Information

Below is the link to the electronic supplementary material.

Supplementary Material 1

## Data Availability

The datasets used in this analysis are publicly available from [https://www.ibge.gov.br/estatisticas/sociais/saude/9160-pesquisa-nacional-de-saude.html](https://www.ibge.gov.br/estatisticas/sociais/saude/9160-pesquisa-nacional-de-saude.html).

## Abbreviations

LMICs: Low and middle-income countries
PNS: Pesquisa Nacional de Saúde
IBGE: Instituto Brasileiro de Geografia e Estatística
FHT: Family health teams
FHS: Family Health Strategy
