# Supplementary material for: Racial inequalities in access to healthcare services in Brazil (2019): a decomposition analysis
Source: BMC Health Serv Res. 2025 Dec 5;25:1573. doi: 10.1186/s12913-025-13527-6 (PMC12681149; doi:10.1186/s12913-025-13527-6)

**Table A1. The procedure for creating the main dependent variables**

| **Variable** | **Variables constructions** | |
| --- | --- | --- |
|  | **Questions and response options in parentheses** | **Values** |
| Unmet need for health care | In the past two weeks, did ___ seek any place, service, or healthcare professional for health-related care (Yes, No, Ignored) | Yes = 1;  No = 0 |
|  | (If yes): The first time you sought healthcare for this reason in the past two weeks (Received care; Scheduled for another day/location; Did not receive care; Ignored) | Received care = 0;  Scheduled for another day/location = 1;  Did not receive care = 1 |
|  | (If no): In the past two weeks, why did ___ not seek healthcare services (No need; Didn't have money; The location was too far or had transportation difficulties; Incompatible hours; Care was very slow; The facility didn't have a suitable specialist; Thought they didn't have the right; No one to accompany them; Didn't like the professionals at the facility; Health services strike; Other reason) | No need = 0;  All other reasons = 1 |
| Unmet need for medication | During this healthcare visit, was any medication prescribed (Yes, No, Ignored) | Yes = 1;  No = 0 |
|  | Did __ manage to obtain the prescribed medications? (Yes, all; Yes, some; No, none; Ignored) | Yes, all = 0;  Yes, some = 1;  No, none = 1 |
| Unable to obtain services last time that sought healthcare | The last time ___ sought healthcare for this reason in the past two weeks, were they attended to? (Yes, No, Ignored)) | Yes = 0;  No = 1 |
| People diagnosed with a chronic disease who have not received medical care in the last year | Has a doctor ever diagnosed you with a chronic disease, physical or mental, or a long-term illness (lasting more than 6 months)? | Yes = 1;  No/Ignored/  Not applicable = 0 |
|  | When did ____ last see a doctor? (Up to 1 year; More than 1 year to 2 years; More than 2 years to 3 years; More than 3 years; Never went to a doctor; Ignored) | If "Up to 1 year" = 0;  Other responses = 1 |

**Figure A1 - Levels of access across four dimensions by racial groups: Black *vs.* Pardo**


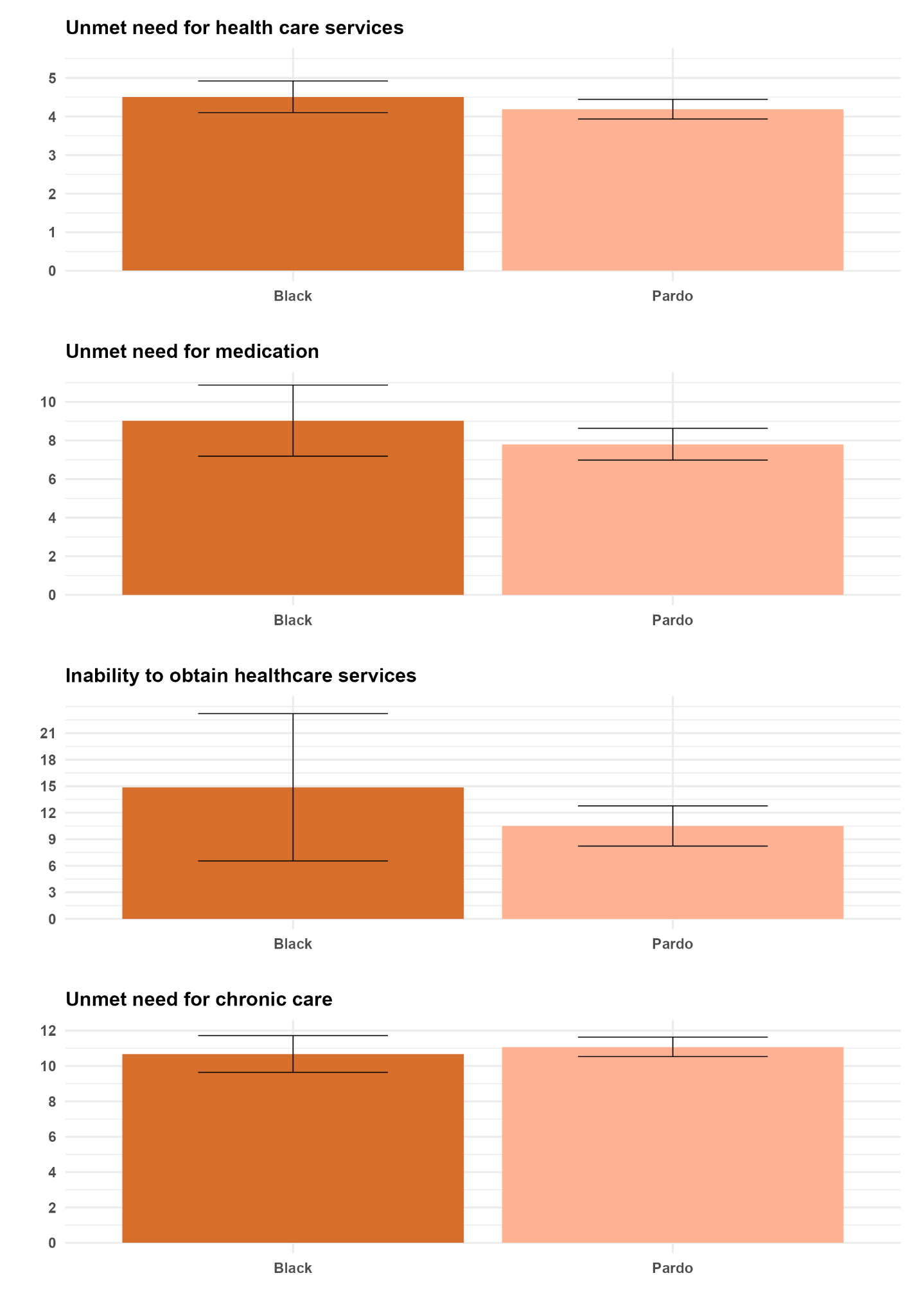


**Figure A2 - Oaxaca-Blinder decomposition for Black *vs.* Pardo**


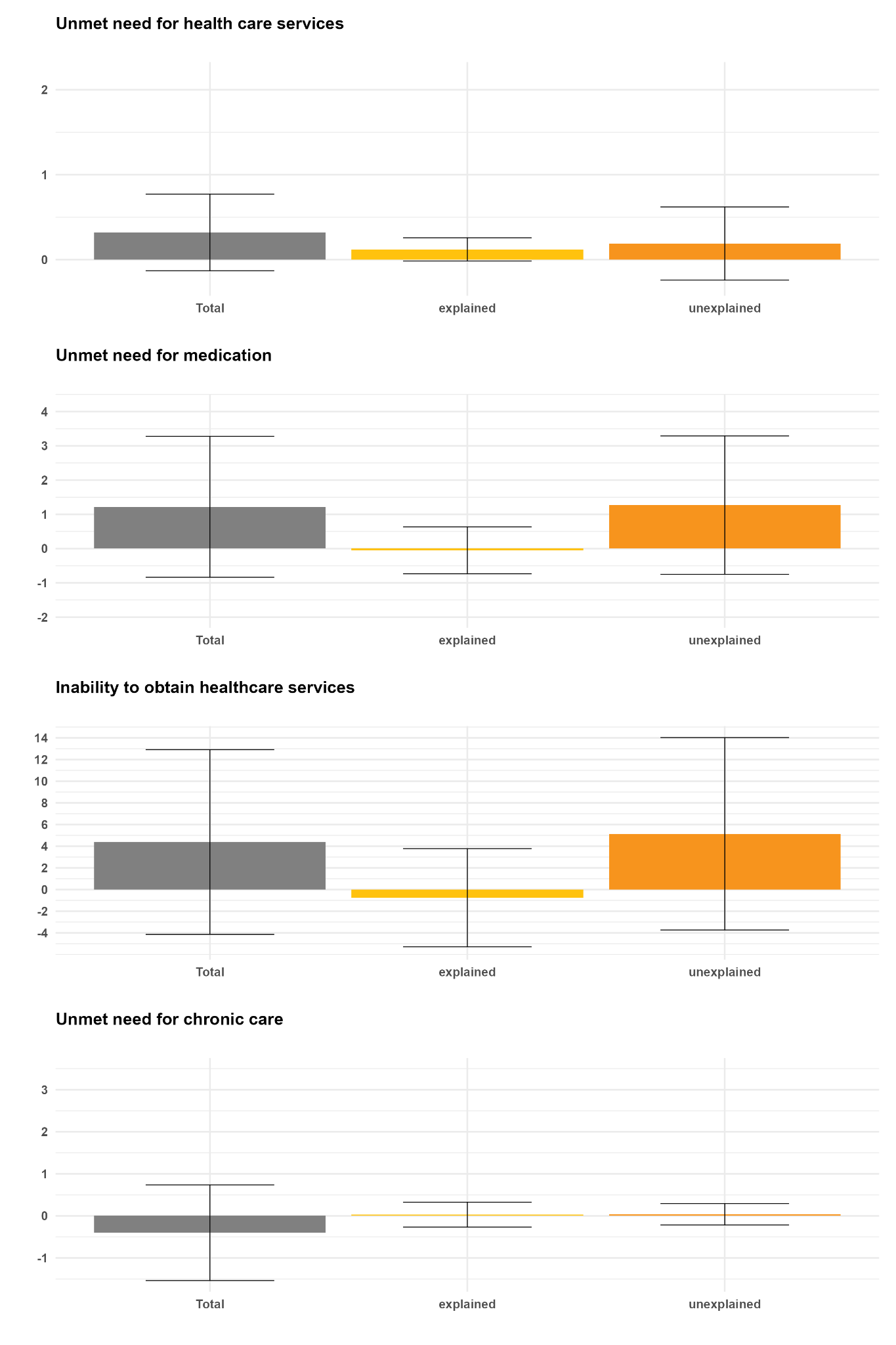


**Figure A3 - Contributions to total difference of the explained part: Black *vs.* Pardo**

##
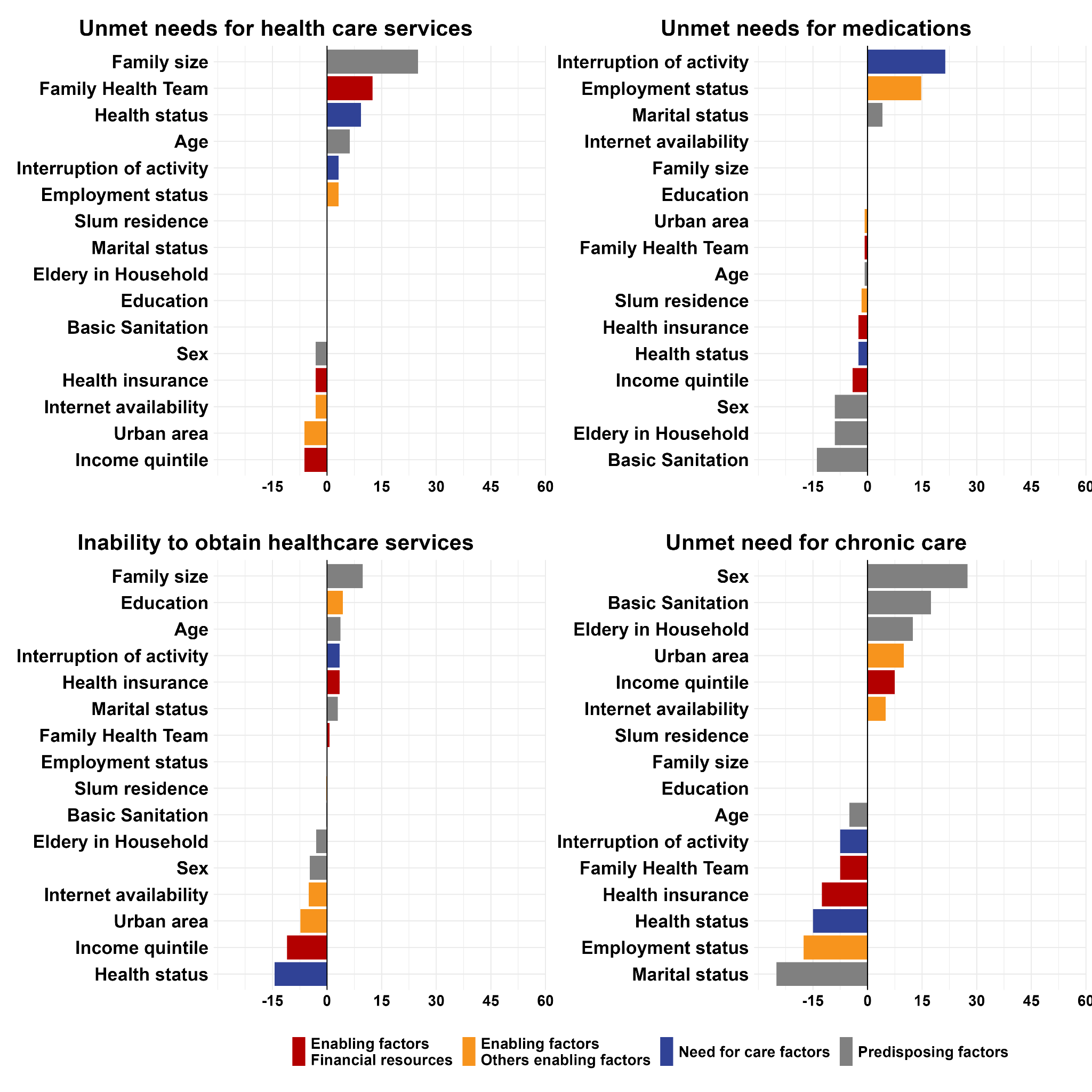

Supplement: Supplementary file 1 — Supplementary Material 1 [file 12913_2025_13527_MOESM1_ESM.docx]
